# Identification of potential plasma biomarkers in early-stage nasopharyngeal carcinoma-derived exosomes based on RNA sequencing

**DOI:** 10.1186/s12935-021-01881-4

**Published:** 2021-03-31

**Authors:** Wei Zheng, Wangzhong Ye, Zijie Wu, Xinyi Huang, Yuanji Xu, Qinyan Chen, Zhizhong Lin, Yanyu Chen, Penggang Bai, Chuanben Chen

**Affiliations:** 1 Department of Radiation Oncology, Fujian Medical University Cancer Hospital, Fujian Cancer Hospital, No. 420, Fuma Road, Fuzhou, 350014 Fujian People’s Republic of China; 2 Fujian Medical University, Fuzhou, Fujian People’s Republic of China; 3 School of Nuclear Science and Technology, University of South China, Hengyang, Hunan China

**Keywords:** Nasopharyngeal carcinoma (NPC), Exosomes, Gene Expression Omnibus (GEO), Bioinformatics analysis, Kyoto encyclopedia of genes and genomes (KEGG)

## Abstract

**Background:**

Early diagnosis of nasopharyngeal carcinoma (NPC) is vital to improve the prognosis of these patients. However, early diagnosis of NPC is typically challenging. Therefore, we explored the pathogenetic roles and associated mechanisms of exosomes in plasma of patients with early-stage NPC.

**Methods:**

Exosomes in plasma were extracted by ultra-high-speed centrifugation. Western blot and transmission electron microscopy (TEM) were used to verify the purity of exosomes. The sequencing data (6 plasma samples from healthy volunteers vs. 6 NPC plasma samples) were analyzed by principal component analysis (PCA), DESeq2, gene ontology (GO), Kyoto encyclopedia of genes and genomes (KEGG), and TargetScan. The differentially expressed miRNAs (DEmiRNAs) were obtained from the dataset (GSE118720) downloaded from the Gene Expression Omnibus (GEO) repository. Additionally, the datasets downloaded from the GEO database (GSE12452, GSE13597, GSE53819, GSE64634) were used to predict the target genes and functions of hsa-miR-1301-3p. qPCR was applied to verify the differences in the expressions of hsa-miR-1301-3p between 10 normal plasma and 10 NPC plasma samples.

**Results:**

Western blot, TEM, and Nanoparticle Tracking Analysis showed adequate purity of the extracted exosomes. RNA-seq analysis revealed 21 upregulated miRNAs, and 10 downregulated miRNAs in plasma exosomes of early-stage NPC patients. GO analysis showed that the target genes of DEmiRNAs were mainly enriched in DNA synthesis and transcription regulation. KEGG analysis revealed that DEmiRNAs were mainly enriched in PI3K-Akt and MAPK signaling pathways. Moreover, the expression of hsa-mir-1301-3p was verified to be significantly upregulated in enlarged samples of plasma exosomes.

**Conclusions:**

We identified several DEmiRNAs extracted from tumor-derived exosomes between normal plasma and early-stage NPC plasma. Bioinformatics analyses indicated that these DEmiRNAs may be related to NPC development. Our study may provide novel insights into underlying biomarkers and mechanisms of plasma exosomes in early-stage NPC.

**Supplementary information:**

The online version contains supplementary material available at 10.1186/s12935-021-01881-4.

## Background

Nasopharyngeal carcinoma (NPC) is a malignant cancer that shows wide variability in geographical distribution. The condition is highly prevalent in Southeast Asia, especially in the Guangdong Province of China [1]. In 2018, an estimated 129,079 new cases of NPC were diagnosed across the world, accounting for 0.7% of all carcinomas [2]. The reported 5-year overall survival (OS) rate of patients with early-stage NPC is 92.8–98.1% while the 5-year OS rate of patients with advanced NPC is 68.9–78.6% [3, 4]. Unfortunately, more than 70% of NPC patients are diagnosed at an advanced stage [5, 6]. Therefore, early diagnosis of NPC is vital to improve the prognosis of these patients. Currently, nasopharyngeal biopsy and imaging examination are the main modalities for the diagnosis of NPC. However, patients with early-stage NPC are typically asymptomatic and are unlikely to be subjected to these investigations; moreover, biopsy is an invasive procedure. Consequently, use of routine screening methods may not be very effective in facilitating early diagnosis of NPC in clinical practice [7]. Therefore, unraveling the mechanisms of the occurrence and development of early-stage NPC and identification of potential molecular biomarkers for early diagnosis of NPC are key research imperatives.

Several risk factors have been implicated in the pathogenesis and progression of NPC, such as Epstein-Barr virus (EBV) infection, genetic susceptibility, and environmental factors [8, 9]. Several studies have indicated that EBV DNA in plasma could be regarded as a biomarker for the early diagnosis of NPC [10, 11]. However, there is no robust evidence supporting its role as a biomarker. Moreover, use of different polymerase chain reaction methods may yield inconsistent results. Different standards for detection tend to limit the clinical application of plasma EBV-DNA [12]. Exosomes contain proteins, nucleic acids (DNA, mRNA, and miRNA), lipids and metabolites [13]. Exosomes have been shown to regulate tumor-induced immune response and promote tumor progression as well as metastasis [14, 15]. Exosomes extracted from breast cancer and prostate cancer cells were shown to induce tumors by transferring miRNAs [16, 17]. miR-200 extracted from exosomes in metastatic breast carcinoma cells promoted epithelial-mesenchymal transformation (EMT) and metastasis of breast cancer cells [18].

In the contemporary literature, an increasing number of studies have investigated the role of exosomes in NPC diagnosis. David et al. found that exosomes extracted from NPC may regulate the tumor micro-environment and affect the growth of adjacent cells through the intercellular transfer of EBV latent membrane protein 1 (LMP1), signal molecules, and viral miRNAs [19]. miR-34c in exosomes was found to inhibit the malignant behavior and radiation resistance of NPC by blocking the EMT process [20]. However, just a few studies have focused on the early-stage NPC, and no reliable biomarkers for the early diagnosis of NPC have been identified. To the best of our knowledge, this is the first study to explore the underlying plasma biomarkers in early-stage NPC exosomes.

The purpose of our study was to explore the underlying mechanism of early-stage NPC and to identify potential markers for early diagnosis of NPC. In the present study, we identified the differentially expressed genes (DEGs) in the exosomes of early-stage NPC. Using the Gene Expression Omnibus (GEO) and the Cancer Genome Atlas (TCGA) databases, bioinformatics analyses were performed to identify the differentially expressed miRNAs (DEmiRNAs) and uncover the potential mechanisms of miRNAs in NPC. Finally, the DEmiRNAs were verified by plasma samples collected at our hospital. Our study may provide novel insights into the early diagnosis of NPC.

## Materials and methods

### Specimen collection

Blood samples were collected from 16 patients with early-stage NPC who were treated at our hospital between November 2018 and September 2019. Plasma samples from 16 age- and sex-matched healthy donors (8 men and 8 women; median age: 50 years) were also collected. In line with the eighth edition of the International Union Against Cancer/American Joint Committee on Cancer guidelines for NPC [21], 7 patients had stage I NPC and 9 patients had stage II NPC. The ethics committee of our hospital approved the use of human tissue specimens in this work (project ethics number: SQ2019-018-01). Six pairs of blood samples were used for the extraction of plasma exosomes, and the other ten pairs of blood samples were used for further sample verification.

### Isolation and identification of exosomes

Melt the samples at 37 °C at medium speed, and move the samples to a new centrifugal tube with centrifugation at 2000*g* at 4 °C for 30 min. Afterwards, carefully move the supernatant to a new centrifugal tube with centrifugation again at 10,000*g* at 4 °C for 45 min to remove the large vesicles. The supernatant was filtrated by a 0.45 μm filter membrane and the filtrate was collected. The filtrate was moved to a new centrifugal tube, and an overspeed rotor was selected for centrifugation at 4 °C and 100,000*g* for 70 min. The supernatant was removed and resuspended with 1 × PBS precooled by 10 mL. The overspeed rotor was selected and centrifuged again at 4 °C for 100,000*g* for 70 min. The supernatant was removed, and 100 μL precooled 1 × PBS was used for resuspend.

Dilute 20 μL exosomes to 90 μL, and add 20 μL fluorescently labeled antibodies (CD9, CD81, IgG) to 30 μL diluted exosomes, respectively. Then mix well, and incubate for 30 min at 37 °C without light. 1 mL precooled PBS was then added, an overspeed rotor was selected, and the rotor was centrifuged at 4 °C for 110,000*g* for 70 min. The procedure was repeated twice. The supernatant was carefully removed and resuspended with 1 × PBS precooled by 50 μL. Finally, the protein index results were detected by nanoflow cytometry measurement(NFCM).

Exosomes were mixed with an equal volume of 4% paraformaldehyde. Next, 10 µL specimens were moved onto Formvar/carbon-coated nickel grids. Subsequently, the membranes were adsorbed for 20 min at room temperature (RT). After washing and fixating using 50 µL 1% w/v glutaraldehyde, specimens were contrasted with 4% w/v uranyl acetate (UA) for 5 min and embedded on ice in a mixture of 4% UA and 2% methylcellulose (100:900) solution for 10 min. The excess liquid was sucked dry and the grill was dried by air. The shape and size of exosomes were checked by transmission electron microscopy (TEM) (JEM-2100 JEOL, Tokyo, Japan).

### RNA extraction and qualification

Total RNA was isolated from the exosomes of early-stage NPC plasma and normal plasma using TRlzol reagent (Invitrogen, South San Francisco, CA, USA) and treated with RNase-free DNase I to remove genomic DNA. RNA degradation and contamination were assessed on 1% agarose gels. The purity, concentration, and integrity of the RNAs were checked using the NanoPhotometer^®^ spectrophotometer (Implen, CA, USA), Qubit^®^. RNA Assay Kit in Qubit^®^ 2.0 Fluorometer (Life Technologies, CA, USA),degradation and RNA Nano 6000 Assay Kit with the Agilent Bioanalyzer 2100 system (Agilent Technologies, CA, USA). All procedures were conducted according to the manufacturer’s instructions.

### Library Preparation and Sequencing

The entire 3 mg total RNA of each sample was divided into aliquots and a small RNA (sRNA) library prepared. The NEBNext Multiplex sRNA Library Prep Set for Illumina (New England Biolabs, Ipswich, MA, USA) was applied to generate the sequencing library. The index code was added to assign a sequence to every specimen. TruSeq SR Cluster Kit v3-cBot-HS (Illumina, San Diego, CA, USA) was applied to cluster index-coded specimens. The libraries were sequenced on an Illumina Hiseq 2000 machine to generate 50 bp single-end reads. The entire process of sequencing was carried out at Shanghai Biotechnology Co. Ltd.

### Data processing

First, the adaptors and low-quality reads (Q30 < 80%) and low complexity reads were removed from the raw reads (unfiltered total reads obtained from the Hiseq 2500) using Fastp (version 0.20.1) and in-house perl scripts; the remaining reads were regarded as clean reads. Contaminants, the reads < 5 nt and trimming the adapters were filtered from these clean reads to obtain the valid reads. The effective reads were used to calculate length distribution and base preference. Moreover, the effective reads were aligned against a reference human genome (GRCh38) using Bowtie. Subsequently, the effective reads were aligned against Rfam (version 14.2) using Bowtie tools to annotate the rRNA, snRNA, snoRNA, sRNA, cis-reg, miRNA, and tRNA species. In the end, the unannotated reads were applied to predict new miRNAs using the miRDeep* software (Version v38). A new GTF file was constructed using the information of the novel miRNAs and the gff3 file in the miRBase. Subsequently, HTSeq v0.6.1 was applied to measure the reads mapped to every miRNA.

### DEmiRNA

The expressions of miRNAs were measured as transcripts per million reads (TPM) by the criteria: Normalized expression = miRNA read count × 1,000,000/lib size (libsize is the sum of miRNA read count). The DESeq2 R package (1.26.0) was used to identify the DEmiRNAs between NPC and normal group. P-values were adjusted for multiple testing by DESeq2 default adjustments. The criteria of DEmiRNAs were *P*-values less than 0.05 and fold change more than 2.

### Target prediction, GO, and KEGG

The potential target genes of DEmiRNAs were obtained from TargetScan (http://www.targetscan.org). TargetScan predicts targets of miRNAs by looking for sites that match the seed region of every miRNA. GO and KEGG analyses were applied to investigate the functions of target genes for the DEmiRNAs by the Database for Annotation, Visualization and Integrated Discovery (DAVID, version 6.8) (http://david.abcc.ncifcrf.gov/) and KOBAS software, respectively. GO and KEGG term with a *P*-value less than 0.05 were considered as significantly enriched by target genes.

### The mRNA-miRNA network construction

Based on the predicted binding relationship between miRNAs and mRNAs, the mRNA-miRNA networks were constructed using Cytoscape v3.7.0 software (http://www.cytoscape.org/).

### Quantitative polymerase chain reaction assay

The hsa-miR-1301-3p expression was quantified by qPCR. In brief, total RNAs were extracted from plasma exosomes obtained from early-stage NPC patients and normal donors using TRIzol reagent (Invitrogen; Carlsbad, CA, USA) and transcribed into cDNA using the RevertAid First Strand cDNA Synthesis Kit (Thermo; Waltham, MA, USA). qPCR assay was conducted by the SYBR Green qPCR Master (Roche). The cel-miR-39 was regarded as external control for quantification of the expressions of miRNAs. All determinations were performed in triplicate. Statistical analysis was performed by the SPSS 22.0 statistical software (SPSS, Inc.; Chicago, IL, USA). The Graph Pad Prism 8 drawing software (GraphPad; La Jolla, CA, USA) was used for generating graphs. Between-group differences were assessed using the Student’s t-test. *P*-values less than 0.05 were considered indicative of statistical significance.

### GEO data processing

The Limma package [22] was used to conduct the normalization and logarithmic transformation with base 2; the DEGs between NPC and normal specimens were identified by Limma. The genetic integration of DEGs screened from the six sets of data was performed using an R package “RobustRankAggreg” [23] according to a robust rank aggregation (RRA) method,which is based on the null hypothesis of irrelevant inputs and identifies genes that consistently rank better than expected.The criterion was as follows: P-value < 0.01.

## Results

### Isolation and characterization of exosomes

Exosomes are microvesicles (length: 30–120 nm) that can transport miRNAs, mRNAs and proteins, which are essential for the cell to cell communication. Exosomes in plasma were extracted by ultra-high-speed centrifugation. Several methods were applied to confirm that the substances isolated were exosomes as recommended in the minimal information for studies of extracellular vesicles 2018 (MISEV2018).

**Fig. 1:**
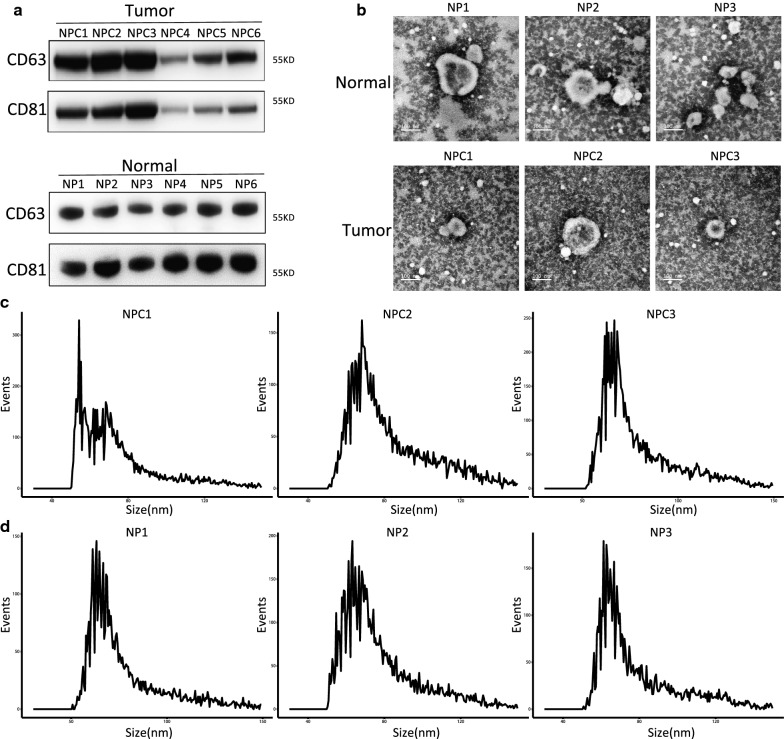
Extraction and identification of exosomes. **a** Results of Western blotting showing the expressions of CD81 and CD63 in the particles. **b** TEM images showing the spherical morphology of the isolated particles (bar = 100 nm). **c**, **d** Size distribution of Exosomes as detected by Nanoparticle Tracking Analysis (NTA). **c** Represents three NPC samples, and **d** represents three normal samples

Western blot was used to determine the expressions of two exosome markers CD81 and CD63. We found abundant expressions of both CD81 and CD63 (Fig. 1a). These results indicated the high efficiency of ultra-high-speed centrifugation in extracting exosomes. Subsequently, TEM analysis revealed round particles with a diameter of approximately 100 nm, which corresponded to the unique characteristics of exosomes (Fig. 1b). Besides, the size of exosomes was checked using NFCM. We noticed that the plasma exosomes were round vesicles ranging from 50 to 150 nm (Fig. 1c, d). The concentration of these particles was further verified by NFCM (Additional file 1: Fig. S1). The results showed that the plasma exosomes were pure enough for further exploration.

### Deep sequencing data

To verify the miRNA expression profile during the progression of NPC, 12 small RNA libraries (6 control and 6 NPC) were constructed for deep sequencing. After removal of poor-quality raw reads, total clean reads were obtained for subsequent bioinformatics analyses. Additional file 2: Table S1 in the supplemental material shows that the sequence reads were produced from all samples, with the single base sequencing error rate below 1%. Both Q20 and Q30 of each sample were > 90%. No GC bias was found. These clean reads were mapped to Rfam databases (Additional file 3: Table S2). The majority of reads were mapped to miRNAs and rRNAs.

**Fig. 2:**
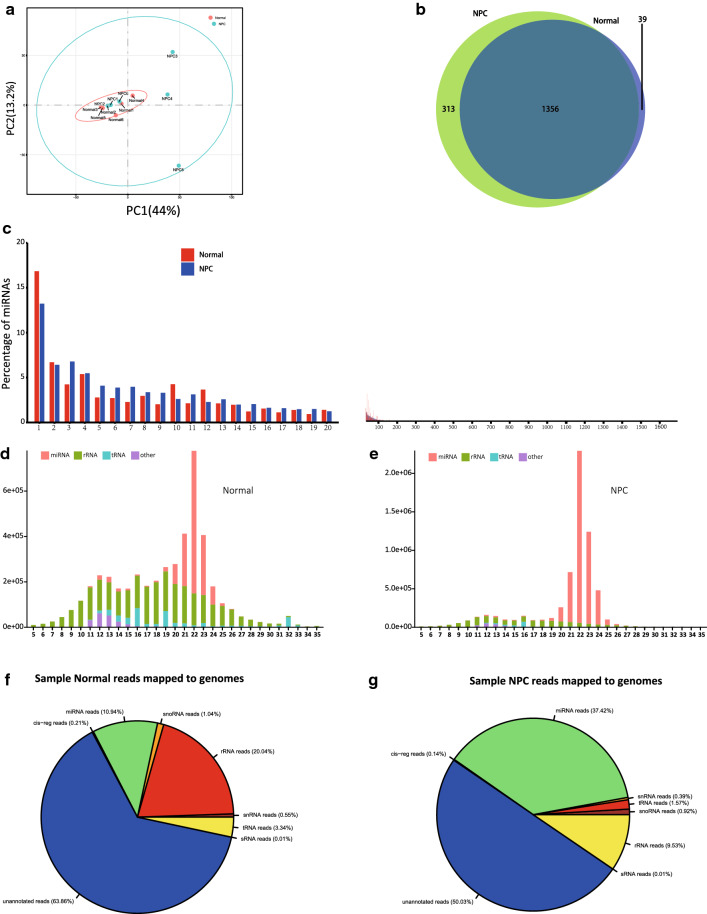
Characterization of exosome miRNAs. **a** Principle component analysis of the total detected miRNAs in Normal and NPC groups (blue dots represent NPC, red dots represent Normal). **b** Venn diagram showing the common and unique miRNAs identified in Normal and NPC groups. **c** Histogram illustrating the percentage of miRNA among the total mapped miRNAs. **d**, **e** Size and frequency distribution of the detected small RNAs (5–35 nt). **f**, **g** Ratio of the percentage of small RNA categories in all reads mapped to Rfam databases

We performed principal component analysis (PCA) of the miRNA expression profiles across 12 specimens to verify the clustering or outliers in the specimen set. Tight clustering was observed between normal samples, but not the tumor ones. Moreover, there was a partial overlap between NPC and normal samples, with NPC samples showing a significantly higher PC1 value than normal. Overall, there was considerable variability with respect to the relative expression patterns across the NPC samples obtained from patients with early-stage NPC (Fig. 2a).

### Profiling the small RNA between control and sample

The types and contents of small RNAs in each sample of NPC and normal were recorded, respectively. The percentage content of small RNAs was calculated by groups (Fig. 2f, g). There was a significant difference in the content of small RNAs of the same kind among the groups, and the contents of miRNAs and rRNAs were the most in both groups.

The abundance of most miRNAs was relatively low in the samples. HTSeq was used to assess the expression of miRNAs in our study. The expression of identified miRNAs was standardized using TPM (transcripts per million; miRNAs were normalized by TPM). The detectable miRNAs identified in at least two samples were retained for subsequent analysis. Consequently, 1669 and 1395 miRNAs were screened; of these, 1356 were common between NPC and normal groups, and 313 and 39 were unique to NPC and normal groups (Fig. 2b). On further analysis of length distribution, the lengths of reads in all samples mostly ranged from 5 to 35 bp. The distribution of the length of small RNAs is depicted in the cluster shaped bar chart (Fig. 2d, e). The annotated sRNA size distribution peaked at 21 nt and 22 nt; 21-22 nt long sequences are regarded as the length of miRNAs in animals. Similar to the data shown in Fig. 2F, G, most small RNAs are miRNAs and rRNAs.

It is well known that the miRNA content is generally small; therefore, high expression of miRNA typically has a special function, which can be used as the focus of further research. We performed statistical analysis of the miRNA expression and drew a histogram. Read counts of screened miRNAs ranged from 0 to 355,139, the 20 most abundant miRNAs accounted for approximately 70% of all mapped reads. The remaining miRNAs accounted for less than 30% (Fig. 2c, Additional file 4: Table S3). Since the total amount of miRNA detected in the two groups is quite different, the ranking is based on the sum of the percentage of each miRNA in the two groups.

### DEmiRNAs

**Fig. 3:**
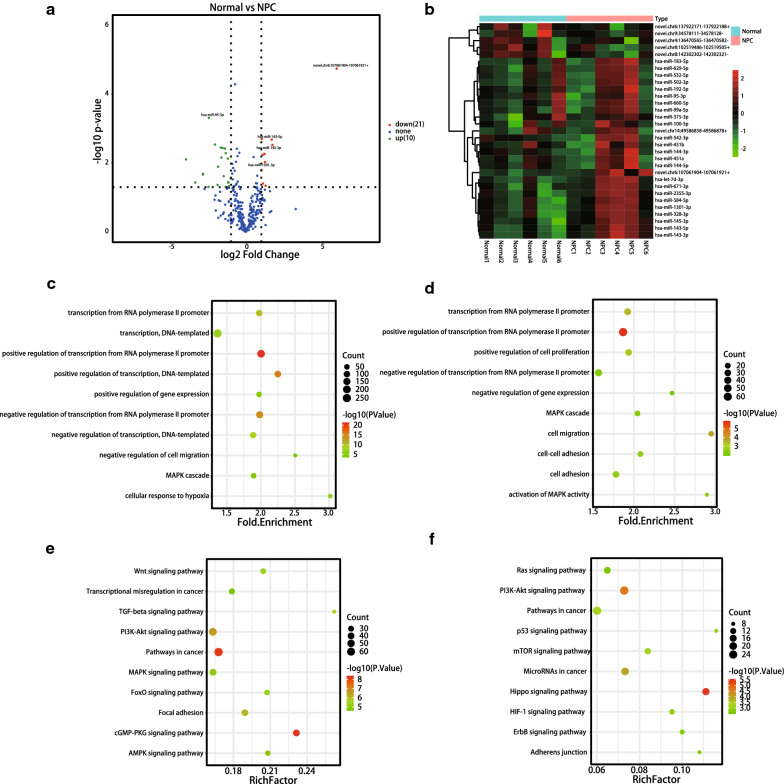
Differential expression and enrichment analysis of exosome miRNAs. **a** Volcano map showing the overall differentially expressed miRNAs (DEmiRNAs) in the two groups. Each point represents a miRNA. Points in green indicate downregulated miRNAs (fold change > 2 and P < 0.05), while points in red indicate upregulated miRNAs (fold change > 2 and P < 0.05). Blue dots indicate miRNAs (fold change < 2 and P > 0.05). **b** Heat map analysis of DEmiRNAs. The color of the heat map indicates the relative miRNA expressions. Green indicates low expression level. Red indicates high expression level. **c** GO analysis for target genes of down-regulated miRNAs. The vertical axis indicates the GO name, the horizontal axis indicates the size of the fold enrichment, the dots represent the number of DEGs in this GO, and the color of the dot relates to different P-value ranges. **d** GO analysis for target genes of upregulated miRNAs. The vertical axis indicates the GO name. The horizontal axis indicates the size of the fold enrichment. Dots represent the number of DEGs in this GO, and the color of the point is related to different P-value ranges. **e**, **f** KEGG for target genes of down-regulated miRNAs (**e**) and up-regulated mi RNAs (**f**). The vertical axis indicates the KEGG name, the horizontal axis indicates the size of the ratio. Dots represent the number of DEGs in this KEGG, and the color of the dot is related to different P-value ranges

Next, we investigated the miRNAs associated with NPC. RNA-seq analysis showed the expression levels of 1708 miRNAs in all samples. Differential expression analysis using DEseq revealed 21 (15 known and 6 novel) down-regulated and 10 (9 known and 1 novel) up-regulated miRNAs (fold change ≥ 2, adjusted P-value < 0.05) (Fig. 3a). A heatmap of DEmiRNAs between NPC and normal groups is shown in Fig. 3b.

### GO, and KEGG of predicted target genes

To investigate the underlying mechanisms of these DEmiRNAs, the target sequences of DEmiRNAs among the groups were predicted using Targetscan. We predicted the target genes for the upregulated and downregulated miRNAs. Next, the target genes were analyzed by GO and KEGG analyses.

GO analysis showed that most of the functional terms were related to the regulation of transcription from RNA polymerase II promoter and MAPK cascade. Figure 3c shows the top 10 downregulated biological processes, while Fig. 3d shows the top 10 upregulated biological processes.

KEGG analysis consistently revealed that most genes targeted by the down-regulated miRNAs were enriched in PI3K-Akt pathway, pathway in cancer, and focal adhesion (Fig. 3e), while the genes targeted by the up-regulated miRNAs were enriched in PI3K-Akt pathway, p53 pathway, and Hippo pathway (Fig. 3f). Among these, the PI3K-Akt pathway has been previously implicated in NPC progression [24, 25]. Our findings are in line with those of previous studies.

### Network analysis of miRNA-mRNA pathway

**Fig. 4:**
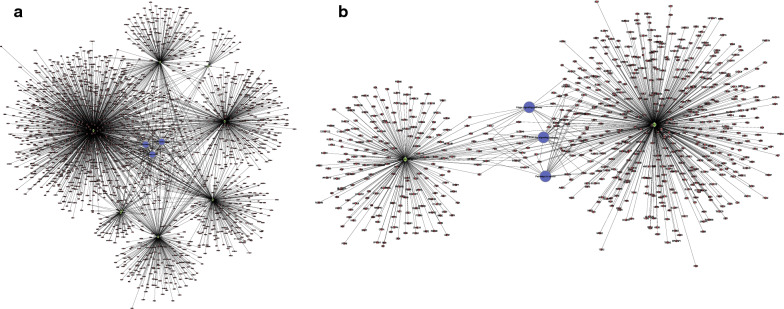
Target gene screening of miRNA. **a** Network between downregulated miRNAs and their potential target genes. **b** Network between upregulated miRNAs and their potential target genes. Diamond, red circles, and blue circles represent miRNA, mRNA, and pathway, respectively

To investigate the functions of miRNAs and the related target genes in NPC, we analyzed the interplay between the miRNAs and the relevant target genes which may help characterize the key regulatory mechanisms of miRNAs. All 1876 genes were predictably targeted by the down-regulated miRNAs, and 697 genes were predictably targeted by the up-regulated miRNAs. Target genes of the DEmiRNAs that were significantly enriched in the KEGG terms were annotated in the network (Fig. 4).

### Important miRNAs related to NPC

**Fig. 5:**
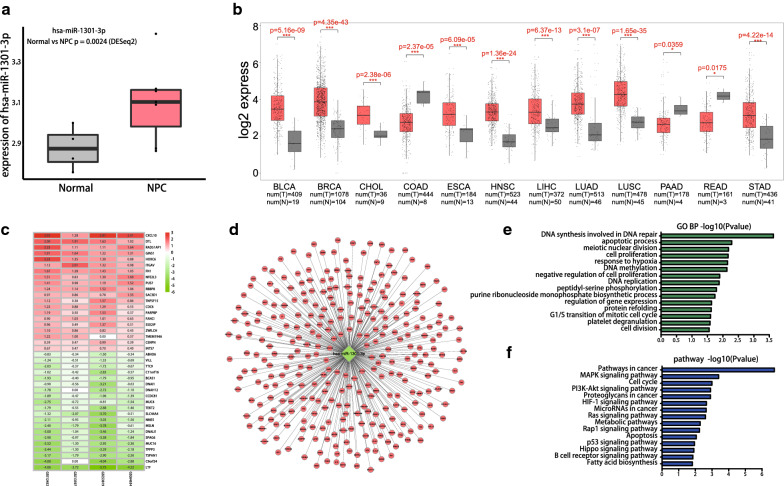
Expression difference and enrichment analysis of hsa-miR-1301-3p. **a** Analysis of the expression of hsa-miR-1301-3p in GSE118720. Red and gray boxes indicate NPC and normal samples, respectively. **b** Analysis of hsa-miR-1301-3p expression level between cancer and paracancerous tissues of different tumors in TCGA(BLCA, BCRA, CHOL, COAD, ESCA, HNSC, LIHC, LUAD, LUSC, PAAD, READ, STAD). The red and gray boxes indicate carcinoma and paracancerous samples, respectively. **c** Heat map of DEGs. Green indicates lower expression level. Red indicates higher expression levels, and white indicates no significant difference in gene expression. Each column indicates a single dataset and every row indicates a single gene. The number in each rectangle refers to the normalized gene expression level. The intermediate colors ranging from green to red indicate the transition from downregulation to upregulation. **d** Interaction network between hsa-miR-1301-3p and its potential target genes. **e** GO terms enriched for target genes of hsa-miR-1301-3p. **f** KEGG enriched for target genes of hsa-miR-1301-3p

Among the DEmiRNAs, we sought to identify the important miRNAs that are related to the progression of NPC. Consequently, we downloaded GSE118720 from the GEO database to study the miRNAs that are differentially expressed between NPC and normal samples. After analyzing the data (Fig. 5a), and then comparing with our data, we finally selected miRNA-1301-3p for further study. There was a significant difference between the two sets of data with respect to the expression of miRNA-1301-3p. We also compared the expression of miR-1301-3p between cancer and paracancerous samples in the context of different tumors in TCGA and drew a box diagram (Fig. 5b). The results showed a significant difference in the expressions of miR-1301-3p between cancer and paracancerous tissues in the context of many tumors.

### Target prediction and functional analysis of hsa-miR-1301-3p

Microarray data (GSE12452, GSE13597, GSE53819, GSE64634) were obtained from the GEO database. RRA was applied to select significantly DEGs in the four datasets (Fig. 5c). Finally, 1406 DEGs were selected. Subsequently, the target genes of hsa-miR-1301-3p were predicted using TargetScan (http://www.targetscan.org/). To improve the reliability of bioinformatics analysis, the overlapping genes were selected for subsequent research (Fig. 5d).

GO and KEGG analyses of the overlapping genes were also performed. The significant GO category involved DNA synthesis in DNA repair, apoptotic process, meiotic nuclear division, and cell proliferation (Fig. 5e). The pathways of target genes that were enriched included MAPK pathway, cell cycle, and PI3K-Akt pathway (Fig. 5f).

### Sample validation

**Fig. 6:**
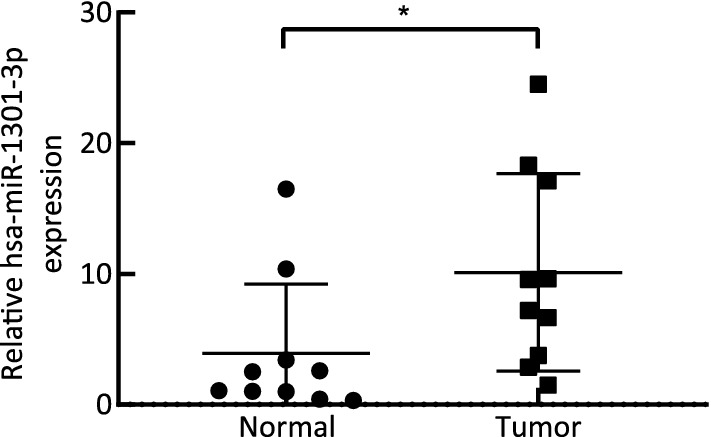
Results of qPCR showing higher hsa-miR-1301-3p expression in NPC samples than in normal samples. *P < 0.05

RT-qPCR was conducted to measure the content of hsa-miR-1301-3p in plasma exosomes of 10 patients with early-stage NPC and normal controls; the results showed significant differences between the two groups (P < 0.05) (Fig. 6).

## Discussion

Early diagnosis of NPC is a key strategy for improving the prognosis of patients [25, 26]. However, accurate early diagnosis of NPC is typically challenging [27]. Therefore, identification of novel diagnostic biomarkers of NPC is a key research imperative. The role of exosomes in carcinogenesis has evoked considerable interest in the context of several tumors such as hepatocellular carcinoma [28], breast cancer [29], and ovarian cancer [30]. The present study is the first to explore potential plasma biomarkers in early-stage NPC exosomes. Exosomes were extracted from the plasma of patients with early-stage NPC at our hospital and healthy donors. We identified 21 downregulated miRNAs and 10 up-regulated miRNAs. The hsa-miR-1301-3p was regarded as a vital (upregulated) DEmiRNAs in early-stage NPC. Moreover, GO analysis revealed that the target genes of DEmiRNAs were mainly enriched in DNA synthesis and transcription regulation. KEGG analysis also showed that the target mRNAs of DEmiRNAs were primarily enriched in the PI3k-Akt and MAPK pathways. Our findings may offer novel insights into the role of exosomes in early-stage NPC.

To obtain the RNAs present in exosomes, exosomes from plasma of early-stage NPC were isolated and characterized. Several methods were used to demonstrate that the exosomes isolated from plasma were indeed exosomes and were pure enough for subsequent experiments. Tumor-derived exosomes represent a liquid biopsy tool with diagnostic and prognostic relevance; in addition, miRNAs present in exosomes may serve as potential biomarkers. Therefore, exosomes may provide novel insights for the early diagnosis of NPC.

We determined the types and contents of small RNAs in plasma samples obtained from patients with early NPC and healthy donors to identify the most abundant small RNAs in both groups. The results showed that miRNAs accounted for most small RNAs in these samples. The 20 most abundant miRNAs accounted for about 70% of all mapped reads. In recent years, the role of miRNAs in carcinogenesis and tumor progression has evoked increasing interest in the context of several cancers, such as lung carcinoma [31], colorectal carcinoma [32], and breast carcinoma [33]. In a previous study, miR-99a was found to inhibit metastasis of NPC by targeting the *HOXA1* gene [34]. Jiang et al. found that EBV miRNA BART2-5p may promote the metastasis of NPC by suppressing the *RND3* gene [35]. Hence, miRNAs represent a focus area for further research.

Identification of the DEmiRNAs is a critical step for investigating the mechanisms by which miRNAs contribute to the initiation of NPC for exploring novel diagnostic biomarkers. In the present study, RNA-seq identified 21 down-regulated and 10 up-regulated miRNAs in NPC. Many of these are associated with cancer; for example, decreased expressions of has-miR-143 and has-miR-145 have also been identified in chronic lymphocytic leukemia [36], lung cancer [37, 38], and colorectal cancer [39], respectively. Daniela et al. demonstrated a positive correlation of miR-183 with cervical cancer [40]. The details of the DEmiRNAs are provided in Fig. 3a and b. Accordingly, we selected these DEmiRNAs for further investigation.

Target genes of these DEmiRNAs were analyzed by GO and KEGG analyses. KEGG analysis showed that the target genes were significantly enriched in the PI3k-Akt and MAPK pathway. The enriched pathways discovered in the present study are mainly involved in cancer progression and metastasis. Several studies have shown that the PI3K-Akt pathway is crucial for the proliferation, migration, and invasion of NPC cells [41, 42]. Downregulation of the *SPEN* gene leads to the inactivation of PI3k-Akt and c-JUN signals, thus inhibiting the migration and invasion of NPC [43]. Zhu et al. revealed that inhibition of autophagy by the *ANXA1* gene may promote the migration and metastasis of NPC by activation of the PI3K-Akt pathway [44].

MAPK plays a vital role in cell proliferation, invasion, and metastasis. Overexpression of miR-4500 may downregulate the *RRM2* gene and inhibit the activation of the MAPK pathway, thereby reducing breast carcinoma cell proliferation, migration, and angiogenesis [45]. In a study by Wan et al., Aur-A was found to promote EMT and invasive growth of NPC through the downstream MAPK pathway [46]. GO and KEGG analyses of target genes of has-miR-1301-3p were largely consistent with the above-mentioned results. Our results indicate that the PI3k-Akt and MAPK pathway play a crucial role in the genesis of NPC. However, the specific underlying mechanisms of the PI3k-Akt pathway and MAPK pathway need to be investigated in further study.

Studies have shown that miRNAs can match their target mRNAs and act on them to inhibit protein translation by degradation of mRNAs [47]. We predicted the target mRNAs of DEmiRNAs by TargetScan. Subsequently, the target mRNAs that were significantly enriched in the KEGG terms were annotated in the network. Furthermore, miRNA-mRNA-pathway networks were constructed via an interplay analysis. Visual networks of NPC were constructed to explore the direct interactions between miRNAs and the corresponding target mRNAs and pathways in early-stage NPC, as well as to investigate the potential regulatory functions of the DEmiRNAs.

To identify important DEmiRNAs related to the oncogenesis of NPC for subsequent research, we analyzed our sequencing data and the GSE118720 dataset downloaded from the GEO database. The findings suggested significant upregulation of the hsa-miR-1301-3p in the plasma exosomes. The results are consistent with high expression of has-miR-1301-3p in the TCGA database. The association of has-miR-1301-3p with proliferation, invasion, and migration of cells has been found in the context of many tumors. In a study by Fang et al., has-miR-1301 was found to inhibit the migration and invasion of HepG2 cells and promote cell apoptosis [48]. However, no studies have investigated the target genes and mechanism of hsa-miR-1301-3p in the context of early-stage NPC. Our study explored the underlying mechanism of hsa-miR-1301-3p using GO and KEGG analyses, which may facilitate a better understanding of the development and progression of early-stage NPC.

Some limitations of our study should be considered. First, the sample size of early-stage NPC patients and normal donors was relatively small. Therefore, larger studies are required to provide more robust evidence. In addition our study was based on bioinformatics analyses. Further experiments are required to provide direct evidence of the mechanisms in early-stage NPC. Furthermore, we did not analyze data pertaining to clinical prognosis of patients with early-stage NPC. Due to the lack of appropriate datasets, we could not investigate the correlation between the DEmiRNAs and the prognosis.

In conclusion, we identified 21 downregulated and 10 upregulated DEmiRNAs in early-stage NPC using bioinformatics analyses. The PI3k-Akt and MAPK pathways might play a vital role in the pathogenesis of NPC. Our findings may provide novel insights into the pathogenetic mechanisms of early-stage NPC and help identify underlying diagnostic biomarkers in plasma exosomes.

## Conclusions

We identified several DEmiRNAs and analyzed the biological processes and signaling pathways of their predicted target genes in early-stage NPC using bioinformatics analyses. Our study may offer novel insights into the potential biomarkers and mechanisms of early-stage NPC. However, further functional experiments are required for in-depth exploration of the molecular mechanisms in early-stage NPC.

## Supplementary information

**Additional file 1: Fig. S1**. Concentration of exosome. **A, B:** Concentration of exosomes as detected by Nanoparticle Tracking Analysis (NTA). **Fig.S1A** represents three NPC samples, while **Fig.S1B** represents three normal samples

**Additional file 2: Table S1.** The sequence reads produced from all samples.

**Additional file 3: Table S2.** The sequence reads of 12 samples were mapped to Rfam database.

**Additional file 4**: **Table S3**. The read counts of the 20 most abundant miRNAs.

## Data Availability

The datasets generated (GSE163867) and/or analyzed during the current study are available in the [GEO] repository, [http://www.ncbi.nlm.nih.gov/geo] [49]. Reference Other datasets used and/or analyzed during the current study are available from the corresponding author on reasonable request.

## Abbreviations

NPC: Nasopharyngeal carcinoma
TEM: Transmission electron microscopy
PCA: Principal component analysis
GO: Gene ontology
KEGG: Kyoto encyclopedia of genes and genomes
DEmiRNAs: Differentially expressed miRNAs
GEO: Gene Expression Omnibus
OS: Overall survival
EBV: Epstein-Barr virus
EMT: Epithelial-mesenchymal transformation
LMP1: Latent membrane protein 1
TCGA: The Cancer Genome Atlas
NFCM: Nanoflow cytometry measurement
PBS: Phosphate buffer saline
RT: Room temperature
sRNA: Small RNA
TPM: Transcripts per million reads
RRA: Robust rank aggregation
MAPK: Mitogen-activated protein kinase
PI3K-Akt: Phosphatidylinositol-3 OH kinase/protein kinase B
