# Supplementary figures and images for: Identification of potential plasma biomarkers in early-stage nasopharyngeal carcinoma-derived exosomes based on RNA sequencing
Source: Cancer Cell Int. 2021 Mar 31;21:185. doi: 10.1186/s12935-021-01881-4 (PMC8011216; doi:10.1186/s12935-021-01881-4)

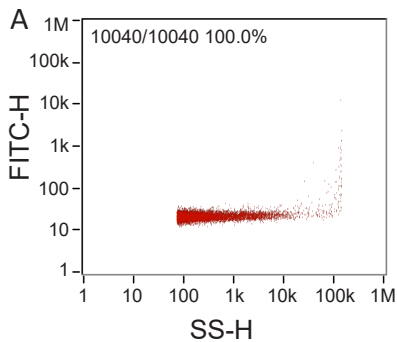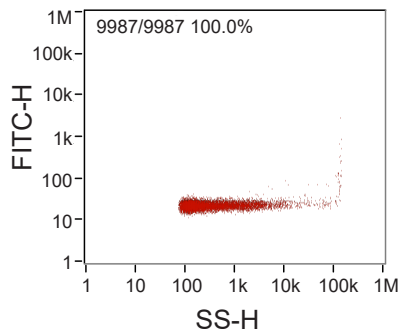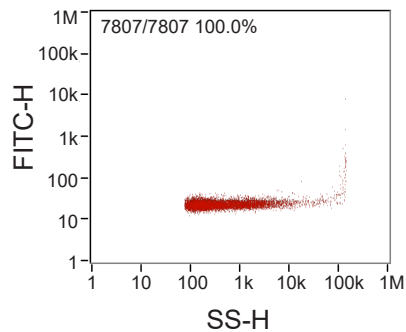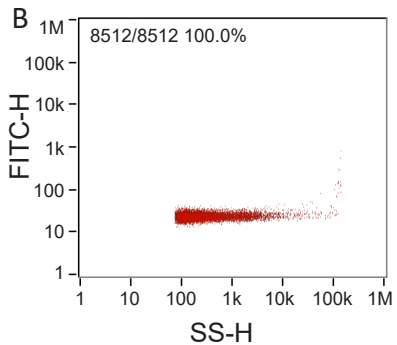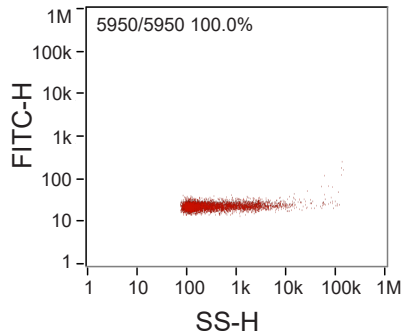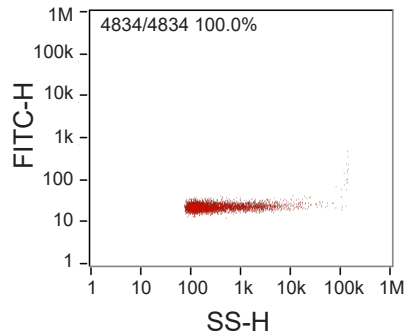

Supplement: Supplementary file 1 — Additional file 1: Fig. S1. Concentration of exosome. A, B: Concentration of exosomes as detected by Nanoparticle Tracking Analysis (NTA). Fig.S1A represents three NPC samples, while Fig.S1B represents three normal samples [file 12935_2021_1881_MOESM1_ESM.pdf]
