# Supplementary material for: Identification of potential plasma biomarkers in early-stage nasopharyngeal carcinoma-derived exosomes based on RNA sequencing
Source: Cancer Cell Int. 2021 Mar 31;21:185. doi: 10.1186/s12935-021-01881-4 (PMC8011216; doi:10.1186/s12935-021-01881-4)
Supplement: Supplementary file 2 — Additional file 2: Table S1. The sequence reads produced from all samples. [file 12935_2021_1881_MOESM2_ESM.docx]

**Table S1: The sequence reads produced from all samples.**

| Sample | clean reads | clean bases | Q20(%) | Q30(%) | NN(%) | GC content(%) |  |  |  |  |
| --- | --- | --- | --- | --- | --- | --- | --- | --- | --- | --- |
| Normal1 | 29722844 | 0.62G | 98.7 | 95.84 | 0 | 55.05 |  |  |  |  |
| Normal2 | 36098457 | 0.76G | 98.23 | 94.84 | 0 | 54.59 |  |  |  |  |
| Normal3 | 34213754 | 0.67G | 98.57 | 95.45 | 0.01 | 58.88 |  |  |  |  |
| Normal4 | 17950365 | 0.37G | 99.19 | 97.28 | 0 | 53.13 |  |  |  |  |
| Normal5 | 35959534 | 0.81G | 99.2 | 97.34 | 0 | 56.53 |  |  |  |  |
| Normal6 | 10890531 | 0.23G | 99.25 | 97.54 | 0 | 51.39 |  |  |  |  |
| NPC1 | 22186155 | 0.44G | 99.17 | 97.13 | 0 | 56.56 |  |  |  |  |
| NPC2 | 24624585 | 0.51G | 99.15 | 97.3 | 0 | 56.77 |  |  |  |  |
| NPC3 | 17518795 | 0.40G | 99.22 | 97.38 | 0 | 50.3 |  |  |  |  |
| NPC4 | 26730124 | 0.62G | 99.16 | 97.37 | 0 | 50.62 |  |  |  |  |
| NPC5 | 21483586 | 0.47G | 97.87 | 93.47 | 0 | 49.71 |  |  |  |  |
| NPC6 | 18323229 | 0.38G | 99.2 | 97.41 | 0 | 56.25 |  |  |  |  |
