# Supplementary material for: Identification of potential plasma biomarkers in early-stage nasopharyngeal carcinoma-derived exosomes based on RNA sequencing
Source: Cancer Cell Int. 2021 Mar 31;21:185. doi: 10.1186/s12935-021-01881-4 (PMC8011216; doi:10.1186/s12935-021-01881-4)
Supplement: Supplementary file 3 — Additional file 3: Table S2. The sequence reads of 12 samples were mapped to Rfam database. [file 12935_2021_1881_MOESM3_ESM.docx]

Table S2: The sequence reads of 12 samples were mapped to Rfam database.

| Sample | rRNA_  Reads | snoRNA_  Reads | snRNA_  Reads | tRNA_  Reads | miRNA_  Reads | sRNA_  Reads | cis-reg_  Reads | annotated_  Reads | Unannotated_  Reads |
| --- | --- | --- | --- | --- | --- | --- | --- | --- | --- |
| Normal1 | 1625708  (12.96%) | 89078  (0.71%) | 48577  (0.39%) | 295169  (2.35%) | 1597837  (12.73%) | 1440  (0.01%) | 18942  (0.15%) | 3676751  (29.3%) | 8871618  (70.7%) |
| Normal2 | 2160511  (14.76%) | 130126  (0.89%) | 77555  (0.53%) | 273508  (1.87%) | 937035  (6.4%) | 2294  (0.02%) | 28037  (0.19%) | 3609066  (24.65%) | 11031106  (75.35%) |
| Normal3 | 2135906  (12.41%) | 208556  (1.21%) | 104346  (0.61%) | 351022  (2.04%) | 576710  (3.35%) | 1966  (0.01%) | 75015  (0.44%) | 3453521  (20.07%) | 13751698  (79.93%) |
| Normal4 | 2221530  (20.75%) | 181017  (1.69%) | 78144  (0.73%) | 175176  (1.64%) | 2443657  (22.83%) | 468  (0%) | 18940  (0.18%) | 5118932  (47.82%) | 5586304  (52.18%) |
| Normal5 | 4893822  (36.19%) | 118936  (0.88%) | 78754  (0.58%) | 210567  (1.56%) | 525024  (3.88%) | 923  (0.01%) | 17211  (0.13%) | 5845237  (43.23%) | 7675733  (56.77%) |
| Normal6 | 2397796  (28.57%) | 76885  (0.92%) | 36019  (0.43%) | 1266719  (15.09%) | 2343933  (27.93%) | 106  (0%) | 4035  (0.05%) | 6125493  (72.99%) | 2267069  (27.01%) |
| NPC1 | 1919555  (16.18%) | 211116  (1.78%) | 112493  (0.95%) | 320920  (2.7%) | 716180  (6.04%) | 2348  (0.02%) | 37325  (0.31%) | 3319937  (27.98%) | 8546203  (72.02%) |
| NPC2 | 1848281  (16.22%) | 177546  (1.56%) | 74730  (0.66%) | 198595  (1.74%) | 716614  (6.29%) | 1549  (0.01%) | 29364  (0.26%) | 3046679  (26.73%) | 8351825  (73.27%) |
| NPC3 | 722939  (6.12%) | 82286  (0.7%) | 25605  (0.22%) | 124835  (1.06%) | 7201273  (60.92%) | 202  (0%) | 7908  (0.07%) | 8165048  (69.07%) | 3656203  (30.93%) |
| NPC4 | 704332  (3.75%) | 90050  (0.48%) | 22896  (0.12%) | 200080  (1.07%) | 9471872  (50.49%) | 167  (0%) | 8025  (0.04%) | 10497422  (55.95%) | 8263468  (44.05%) |
| NPC5 | 1426377  (8.28%) | 73022  (0.42%) | 36156  (0.21%) | 217093  (1.26%) | 10529098  (61.1%) | 191  (0%) | 8051  (0.05%) | 12289988  (71.31%) | 4943526  (28.69%) |
| NPC6 | 868837  (11.5%) | 91986  (1.22%) | 36792  (0.49%) | 169736  (2.25%) | 786414  (10.41%) | 627  (0.01%) | 17805  (0.24%) | 1972197  (26.11%) | 5581572  (73.89%) |
