# Supplementary material for: Identification of potential plasma biomarkers in early-stage nasopharyngeal carcinoma-derived exosomes based on RNA sequencing
Source: Cancer Cell Int. 2021 Mar 31;21:185. doi: 10.1186/s12935-021-01881-4 (PMC8011216; doi:10.1186/s12935-021-01881-4)
Supplement: Supplementary file 4 — Additional file 4: Table S3. The read counts of the 20 most abundant miRNAs. [file 12935_2021_1881_MOESM4_ESM.docx]

Table S3：The read counts of the 20 most abundant miRNAs.

| miRNA | Normal_average | Normal_percentage | NPC_average | NPC_percentage |
| --- | --- | --- | --- | --- |
| hsa-miR-21-5p | 129308 | 0.168497262 | 355139 | 0.132303114 |
| hsa-let-7g-5p | 51457 | 0.067052028 | 172378 | 0.064217521 |
| hsa-miR-191-5p | 32497 | 0.042345837 | 182127 | 0.067849403 |
| hsa-miR-126-3p | 41309 | 0.053828482 | 147298 | 0.054874244 |
| hsa-miR-185-5p | 21363 | 0.027837466 | 109944 | 0.040958424 |
| hsa-miR-30e-5p | 20818 | 0.027127293 | 104257 | 0.038839794 |
| hsa-miR-221-3p | 17532 | 0.022845408 | 106611 | 0.039716751 |
| hsa-let-7i-5p | 22687 | 0.029562729 | 90482 | 0.03370807 |
| hsa-miR-30d-5p | 15500 | 0.020197571 | 88598 | 0.033006207 |
| hsa-miR-451a | 32670 | 0.042571268 | 69999 | 0.026077355 |
| hsa-miR-146a-5p | 16390 | 0.021357303 | 83929 | 0.031266822 |
| hsa-miR-27b-3p | 28103 | 0.036620151 | 61219 | 0.022806463 |
| hsa-miR-26b-5p | 16275 | 0.02120745 | 69361 | 0.025839675 |
| hsa-miR-142-5p | 15089 | 0.01966201 | 53546 | 0.019947971 |
| hsa-miR-223-3p | 9415 | 0.012268396 | 55012 | 0.020494113 |
| hsa-miR-320a-3p | 11816 | 0.015397065 | 43937 | 0.016368244 |
| hsa-miR-25-3p | 8643 | 0.011262426 | 42580 | 0.015862709 |
| hsa-let-7d-5p | 10655 | 0.013884201 | 39794 | 0.014824815 |
| hsa-miR-148b-3p | 7136 | 0.009298701 | 40143 | 0.014954832 |
| hsa-let-7b-5p | 10778 | 0.014044479 | 33459 | 0.012464781 |
